# Climate change impact on water resources availability in the kiltie watershed, Lake Tana sub-basin, Ethiopia

**DOI:** 10.1016/j.heliyon.2023.e13941

**Published:** 2023-02-21

**Authors:** Melsew A. Wubneh, Tadege A. Worku, Bantalem Z. Chekol

**Affiliations:** a Department of Hydraulic and Water Resources Engineering, University of Gondar, Gondar, Ethiopia; b Department of Hydraulic and Water Resources Engineering, Debre Tabor University, Debre Tabor, Ethiopia

**Keywords:** Kiltie, Climate change, Water availability, Ensemble, HBV

## Abstract

Climate change's influence on water resource availability in watersheds must be evaluated to ensure food and water security. Using an ensemble of two global climate models (MIROC and MPI) and one regional climate model (RCA4), the impact of climate change on the availability of water in the Kiltie watershed was evaluated under the RCP4.5 and RCP8.5 scenarios for the year 2040s and 2070s. The flow was simulated using the HBV hydrological model, which needs fewer data and is typically employed in data-scarce settings. The model calibration and validation result, show RVE (relative volume error) of −1.27% and 6.93%, and NSE of 0.63 and 0.64 respectively. Seasonal Water Supply in the Future Under the RCP4.5 Scenario for the 2040s increased between 1.1 mm and 33.2 mm showing maximum incremental in August and a decrease in a range from 0.23 mm to 6.89 mm with a maximum decrease in September. While in the 2070s, water availability increases between 7.2 mm and 56.9 mm, with the largest increases occurring in October and the smallest reductions occurring in July by 9 mm. Future water availability increases under the RCP8.5 scenario during the 2040s period between 4.1 mm and 38.8 mm, with the highest increase occurring in August, and falls between 9.8 mm and 31.2 mm, with the maximum declines occurring in the spring seasons. Water availability in the 2070s, according to the RCP8.5 scenario, increases between 2.7 mm and 42.4 mm with the highest increments in August, and it decreases between 1.8 mm and 80.3 mm with maximum decreases in June. According to this study, climate change would make it easier to access water during the rainy season, necessitating the construction of water storage facilities so that surplus water can be used for dry farming. A watershed-level integrated water resource management strategy should be created quickly as future water supply will decline during the dry seasons.

## 1 Introduction

Temperature and precipitation trends can affect the hydrological cycle's components, as well as the availability of water resources in the watershed or basin [1]. Knowing the existing and future trends in freshwater availability is crucial for adjusting to a changing and unpredictable climate since freshwater availability is essential for food security, human health, ecological health, land use change, economic development, and regional conflict [2]. Hydrological cycle will be accelerated by a warmer climate, modifying rainfall patterns along with the amount and timing of storm runoff [3]. Changes in flow and rainfall within the watersheds could result in a diversified impact on water availability for agricultural, domestic water supply, and hydropower demands. The importance of freshwater is growing due to an increase in worldwide demand for the resource brought on by population expansion. The amount of freshwater that is accessible on earth is unevenly distributed despite the rising demand. Estimates predict that as a result of rising population growth, industrial expansion, climate change, and developing irrigation-based agriculture in the future decades, there will be a significant increase in the demand for water resources to provide enough supplies for human life [4]. In the majority of dry subtropical regions, climate change is anticipated to drastically reduce renewable surface water and groundwater resources. It would also aggravate competition for water among agriculture, ecosystem settlement, industry, energy, and food security [5]. Evidence suggests that the country's mean annual temperature has risen by around 1.3 °C since 1960, or an average rate of 0.28 °C per decade, and that rainfall variability has also increased over time [6]. Four of the nine GCMs examined for the Special Report on Emissions Scenarios A2 scenario showed statistically significant losses in annual streamflow for the 2080–2100 period in the Lake Tana basin (where the Kiltie watershed is located) [7]. Previous research in Ethiopia's upper Blue Nile Basin examined how temperature, hydrology, flow, and water availability were impacted by climate change. For instance Ref. [8], have been evaluated streamflow implications of climate change in the upper Gilgel Belles watershed [9], examined how the Gilgel Abay watershed's discharge will be affected by climate change, and [10] studied the effects of climate change and land use changes on the Gumara watershed's hydrological responses [11]. have examined the effects of climate change on the upper Blue Nile Basin's water resources using the A1B scenario, and their findings indicated that precipitation will rise by 7%–48% and streamflow by 21%–97%. Due to climate change, different watershed characteristics result in different flow characteristics and water availability for each individual watershed. Consequently, this study evaluates how climate change will affect the Kilties future access to water which is a tributary of Lake Tana where a variety of socioeconomic activities in the surrounding community relies on it. Although prior research such as [7,8,12,13] has done works on this basin, most of them are based on outdated climate scenarios and a single GCM climate model. They used SRES (special report emission scenario) that the GCM resolution is somehow coarse and mostly dependent on a statically downscaling approach. Examining the amount of the impact will assist the decision makers in creating a water management plan that takes future water supply and demand into account, as the influence of climate change may lower future water availability in the watershed. In this study, an ensemble of GCM models (i.e., MIROC and MPI) were used for a single regional climate model (RCM) of RCPA4 under the most recent representative concentration pathways (RCPs). The median scenario (RCP4.5) and the highest concentration (RCP8.5) under the future time domains of the 2040s and 2070s were examined here. The study selected the worst-case scenarios, RCP4.5 and RCP8.5, in which a significant impact is likely to occur. The study is planned to look at how the RCP4.5 and RCP8.5 scenarios in the 2040s and 2070s time frame will affect the availability of water resources and flow in the Kiltie watershed.

## 2 Materials and methodology

### 2.1 Study area

**Fig. 1:**
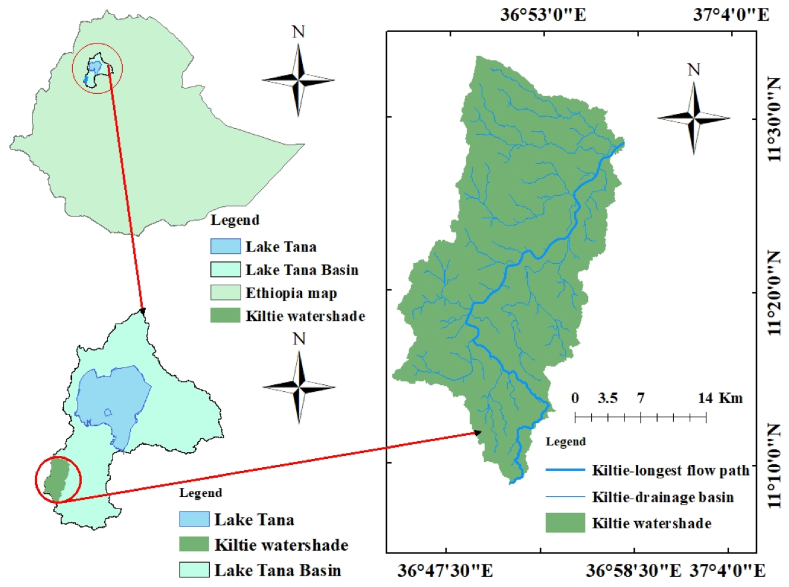
Study area Location.

Geographically, the Kiltie watershed is situated in the Lake Tana sub-basin (Fig. 1). It is a lower tributary of the Gilgel Abay River, which supplies Lake Tana with the majority of its water. The Kiltie River has a 67.3 km length and a total area of about 604 km^2^. The geographical coordinate (UTM) of the area is 1278823 m North and 253009 m east at the outlet point. The watershed has an average slope of 52.4% an annual rainfall of 1568 mm and an altitude of 2268 m.a.s.l. The socioeconomic activities of the people in the watershed are mostly dependent on agriculture, fisheries, and animal husbandry. The mean annual rainfall from the 1988 to 2005 period in the watershed is 1500 mm and the mean monthly maximum temperature is 4.50c.

### 2.2 Methodology

#### 2.2.1 Observed data

**Fig. 2:**
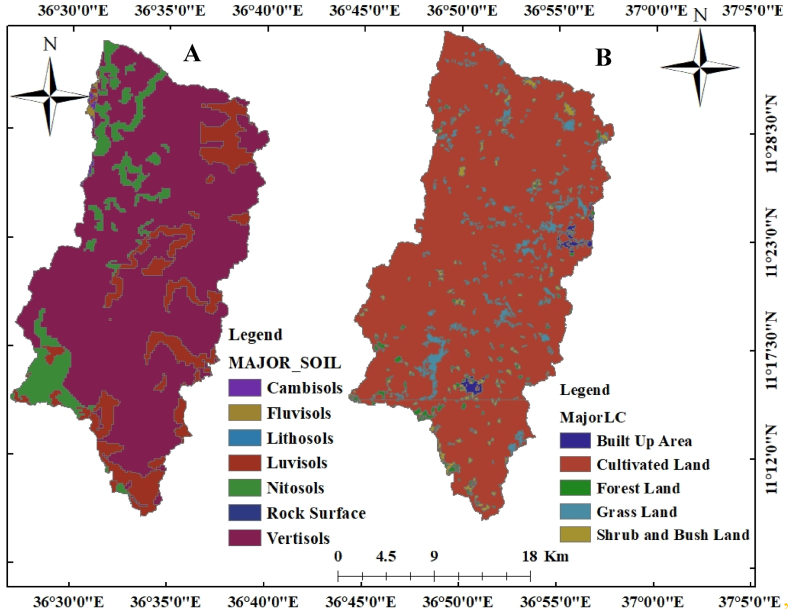
Kiltie watershed soil (A) and kiltie watershed land use land cover (B).

The Bahir Dar National Metrological Agency provided gauge meteorological data from 1976 to 2005 for use in the assessment of how climate change may affect the supply of freshwater. Hydrological data from 1988 to 2005 was made available by the ministry of water, energy, and irrigation (MOWEI) for model calibration and validation. Data on land usage, land cover, and soil were gathered from the Ethiopian Ministry of Water, Energy, and Irrigation (MOWEI) (Fig. 2). demonstrates the watershed's soil and land use and cover. .

#### 2.2.2 Baseline data

Baseline climate data were obtained at CMIP5 under the CORDEX Africa project at ESGF (earth system grid federation portal.) As a baseline, the average data of MIROC and MPI GCM-RCM combinations from the 1976 to 2005 period were used. This aids in the evaluation of the influence on the availability of water resources.

#### 2.2.3 Future time domain climate data

The study used a 0.44° grid of future climate data from the CMIP5 CORDEX project, which was dynamically downscaled using global warming time series from four GCM-RCM combinations with one regional model (RCA4). The water availability under climate change consequences has been examined using RCP4.5, the middle scenario, and RCP8.5, the highest scenario. Even though there are other scenarios, including RCP2.6, RCP4.5, RCP6, and RCP8.5, this analysis only takes RCP4.5 and RCP8.5 into account because they represent the intermediate and highest emission scenarios, respectively. RCP2.6 is the lowest scenario which is less impact full and not considered while RCP6 is somehow similar and considered as an intermediate scenario as RCP4.5. Hence RCP4.5 can be considered as RCP6. RCP8.5 is impact full with a high emission rate. Future climate data has been applied to several periods, such as 2020–2050 (the 2040s) and 2050–2079 (2070s). Each time domain has been considered thirty years, as climate change impact assessment is done using long time series data.

#### 2.2.4 Performance evaluation of climate models

As mentioned by Ref. [14] GCM/RCM model performance based on monthly time series is evaluated using RMSE, percent of bias, correlation, and Nash Sutcliffe Efficiency (NSE) criteria. (1), (2), (3), (4)) depicts RMSE, % of biased, correlation and NSE. Correlation and Nash Sutcliffe Efficiency respectively.

(1) $$RMSE=\sqrt{1/n\sum \left(M-O\right)}$$

(2) $$\%BIASED=\frac{M_{mean}-O_{mean}}{O_{mean}}*100$$

(3) $$r=\frac{rmo}{rm*ro}$$

(4) $$NSE=1-\frac{\left\{\sum_{i=1}^{N}\left(Mi-Oi\right)\right)2}{\sum_{i=1}^{N}\left(Mi-O_{mean}\right)2}$$

**Fig. 3:**
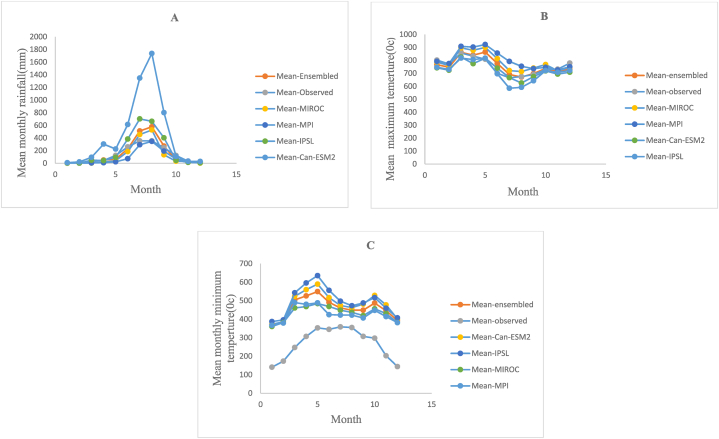
Representation of mean monthly observed rainfall (A), Tmax (B), and Tmin (C) with different GCMs and with their ensemble.

Where rmo is the covariance between the GCM/RCM and observed data, rm and ro are the standard deviations for the GCM/RCM and the ground-observed data, respectively, and n is the number of years taken into consideration in the analysis. M represents data for GCM/RCM time series. O represents the corresponding time series of ground observations. The performance analysis of rainfall shows a correlation(r) greater than 0.7 for all four GCM-RCM combinations. The NSE value for MPI and MIROC GCM models better represents the observed data as compared with Can-ESM2 and IPSL GCM models. As shown in Fig. 3 When compared to IPSL and Can-ESM2, MPI and MIROC climate models better capture the seasonal variability of the observed data. Maximum temperature best captures the seasonal variability of observed data as compared to the minimum temperature. Hence ensemble rainfall, maximum temperature, and minimum temperatures of the MPI and MIROC models were used for impact analysis. Typically, climate models with the least amount of bias, the lowest RMS error, and the highest correlation (r) have been used to analyze how climate change may affect the availability of water resources performance of four GCMs is shown in Table 1 below. Fig. 3 (A, B, and C) depicts comparison of mean observed rainfall, maximum temperature and minimum temperature with different GCM models and their ensemble value.

**Table 1:** Performance value for MIROC and MPI models (A), IPSL and Can-ESM2 model(B) under the RCA4 regional climate model (RCM).

A	MIROC				MPI			
	r	biased	RMSE	NSE	r	biased	RMSE	NSE
RF	0.9	68	27	0.6	0.9	−43.5	68.7	0.65
T_max_	0.8	33	54	0.8	0.9	51.3	76.2	0.83
T_min_	0.8	212	219	0.7	0.6	227	233	0.75
B	IPSL				Can-ESM2			
	r	biased	RMSE	NSE	r	biased	RMSE	NSE
RF	0.8	69.5	149	0.5	0.8	312.2	534	0.55
Tmax	0.6	−38.3	47	0.64	0.7	−27.7	41.7	0.7
Tmin	0.7	158	170	0.6	0.7	163.7	172	0.68

#### 2.2.5 Biased correction of climate data

**Fig. 4:**
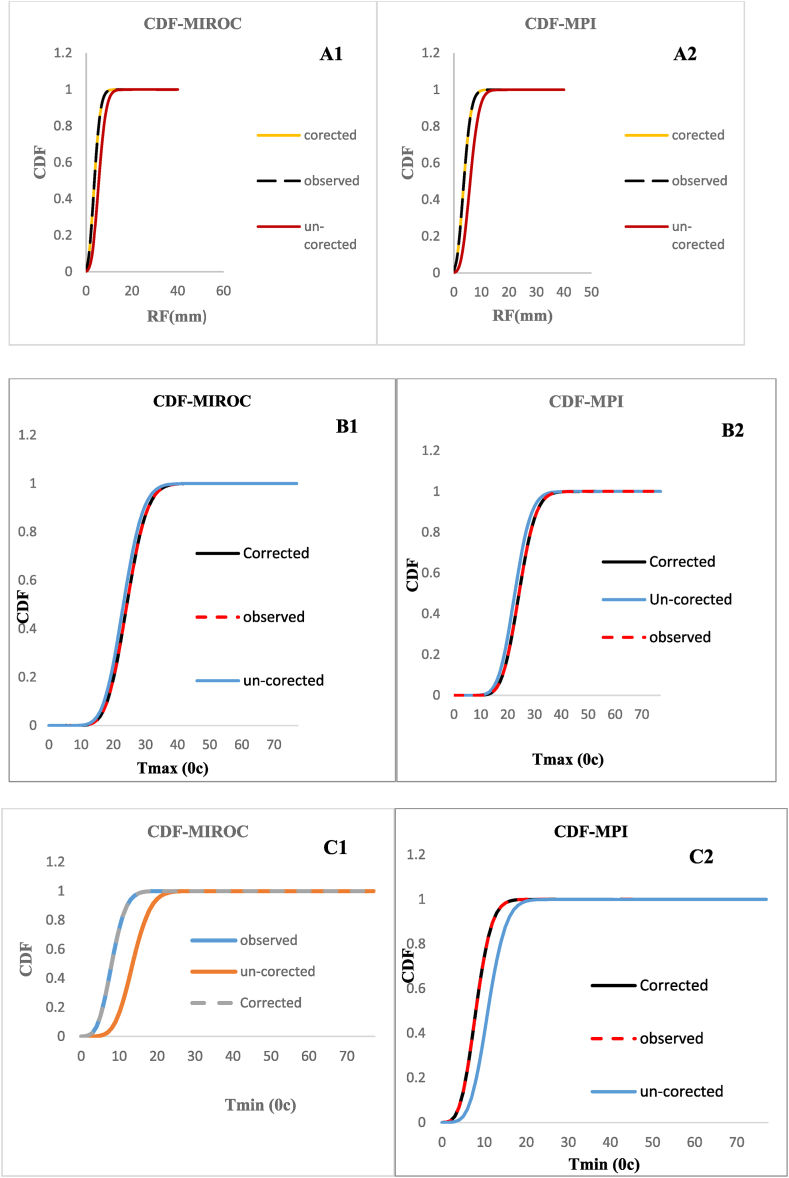
CDF graph for Rainfall (**A1&A2**), Tmax (**B1&B2**), and Tmin (**C1&C2**) of MIROC and MPI GCM models.

As raw GCM-RCM climate downscaled data are by nature subject to biased due to coarser resolution, hence biased correction should be applied to reduced uncertainities made between the ground-observed and GCM/RCM data for further climate change impact assessment. Different bias-correction techniques, such as linear scaling, delta change correction, local intensity scaling of precipitation, power transformation of precipitation, variance scaling of temperature, and distribution mapping (quantile mapping) of precipitation, are frequently used in climate change impact studies [15]. This study employed quintile mapping approach and power transformation to correct temperature and rainfall biases respectively. The Quantile mapping approach works through the principle of normal distribution function. Quantile mapping was employed in this work instead of other bias correction techniques because it uses the transfer function to convert the cumulative distribution of simulated data to the cumulative distribution actual gauge data. This technique can match every statistical moment and capture the progression of the mean and data variability [16]. [17] also evaluate the performance of different biased correction methods and states that quantile mapping can better simulate GCM/RCM model correlated with the ground –observed data during corrections of rainfall and temperature data. Fig. 4(A-C) depicts the climate models MIROC and MPI before and after biased correction using the cumulative distribution function (CDF).

#### 2.2.6 Hydrological modeling

The elements of the watershed's water balance were simulated using the HBV model. A popular conceptual model is the HBV (Hydrologiska Byrns Vattenbalansavdelning)-Light hydrology model. An illustration of a semi-distributed conceptual model is this one. The entire watershed is separated into various sub-watersheds, each of which is further divided into various zones of elevation and vegetation. It functions using evaporation, air temperature, and daily and monthly rainfall data. Snow accumulation is determined using information about air temperature. Equation (5) outlines the general water balance equation that was employed.

(5) P−E−Q=d/dt(SP+SM+UZ+LZ+Lakes)

P refers to precipitation, E to evaporation, Q to runoff, SP to snowfall, SM to soil moisture, UZ to upper groundwater zone, LZ to lower groundwater zone, and lakes to lake volume [18]. Observed daily rainfall, daily temperature, long-term monthly potential evapotranspiration, and daily runoff data are used to calculate runoff [12]. It makes advantage of a warm-up phase, during which the state variables will acquire their proper values in accordance with meteorological data and parameter values.

Even though there are a variety of hydrological models available, the HBV model was chosen because it does not require a lot of data (applicable for the data-scarce area). Previous studies such as [13,19] in the Lake Tana basin have been done using the HBV model and the model performs well. The model's input variables include the mean daily temperature, daily rainfall, spatial information (land use and land cover, DEM), and long-term mean monthly evapotranspiration calculated from the mean daily maximum and minimum temperature.

## 3 Result

### 3.1 Hydrological modeling performance

**Fig. 5:**
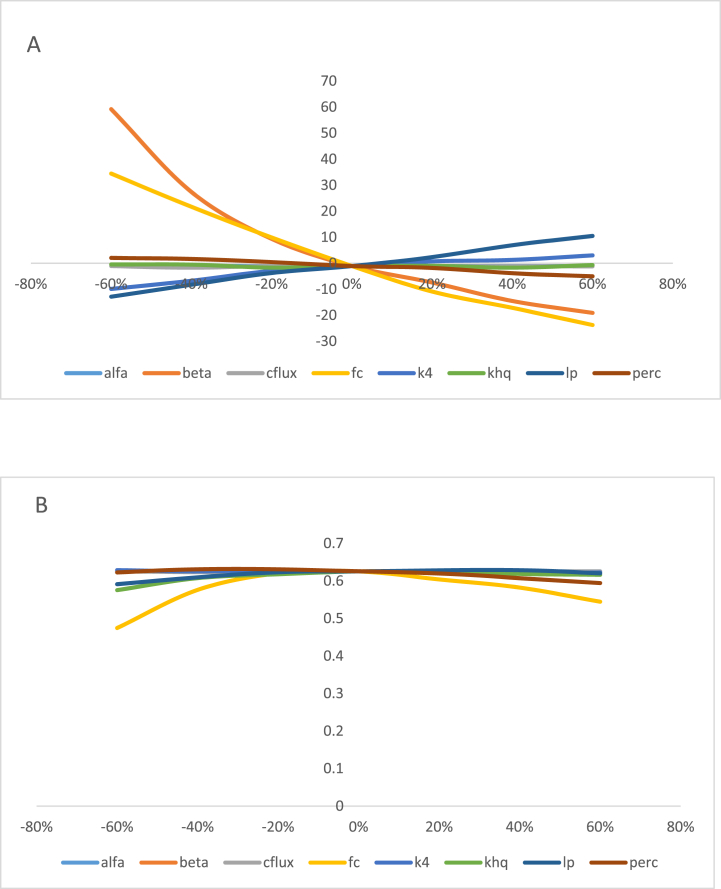
Graphical interpretation of sensitivity analysis for RVE (A) and NSE (B) for kiltie watershed.

**Sensitivity**: The HBV model sensitivity analysis was done by computing RVE and NSE through changing the model parameter with specified common interval to both directions (left and right) from the origin (0,0). By manually altering the value of one optimized model parameter with a properly restricted range of common intervals at a time, sensitivity analysis for the model was carried out (i.e., both increasing and decreasing to the right and left respectively). For the purposes of this study, the value of each optimized model parameter was increased and decreased up to 60% with a common interval of 20%, and a plot of these increased and decreased values versus the model performance evaluation criteria (i.e., NSE, RVE, or R2) for each model parameter was then made. The parameters with steep slopes were considered to be the most sensitive parameters, and those with gentle slopes were considered to be less sensitive optimized parameters. Based on the result of sensitivity analysis model parameters such as BETA, FC, and LP were more sensitive meaning that a minimum change on it will have significant change on flow. Model parameters like ALFA, CFLUX, K4, KHQ, and PERC are less sensitive indicating that with changing its value by whatever amount could not show significant change on flow. Fig. 5(A, and B) demonstrates the findings from sensitivity analyses performed on the Kiltie watershed for RVE and NSE values, respectively.

**Fig. 6:**
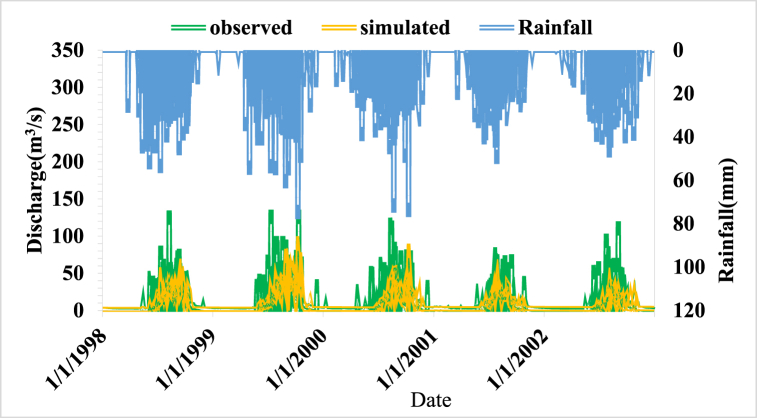
Model calibration results graph (1998–2002).

**Calibration:** NSE greater than 0.6 and an RVE value between −5% and +5% are generally acceptable results for model calibration. For this study, an RVE and NSE value of −1.27% and 0.63 were obtained respectively. As the model performance shows within the acceptable range, it was possible to use the model for further simulations of flow. Fig. 6 shows the calibration model result hydrograph.

The base flow, rising limb, and recession limb of the watershed are well represented in the simulated and observed hydrograph for the optimized value. The simulated discharge is understated while the observed discharge is overstated, as can be seen on the hydrograph in Fig. 6 of this article. This may reflect variations in the area's rainfall, or it may reflect typographical errors in some gauged sites where recorded data indicates a peak value. The overall calibration result displays a satisfactory agreement when compared to the performance evaluation criteria (RVE and NSE).

**Fig. 7:**
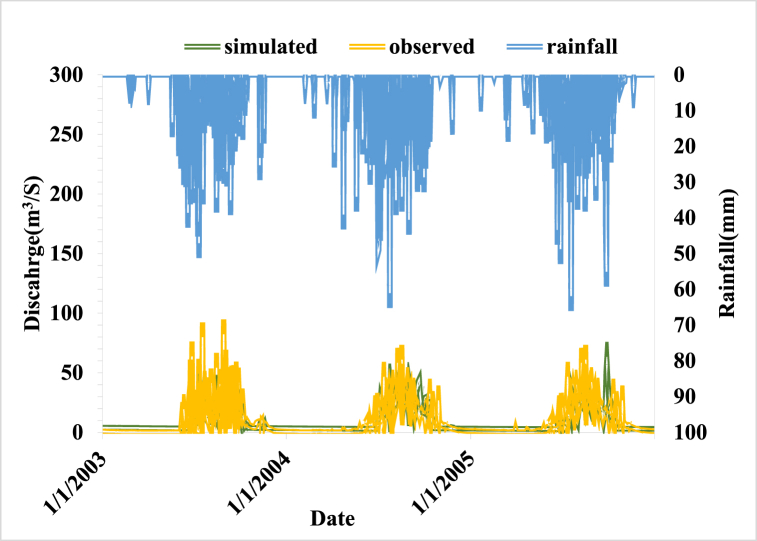
Model validation results graph (2003–2005).

**Validation:** As evidenced by the calibration interval, the model validation outcome in this study performs reasonably. While RVE remained within a reasonable range for Kiltie, NSE value indicated an improvement in model performance. The NSE value for the entire validation findings was larger than 0.6, while the RVE value ranged from −5% to +5% which is in acceptable range. During a validation period, the model results in an NSE value of 0.64 and an RVE value of 6.93% which is in the recommended range. Fig. 7 shows the validation model result hydrograph.

The base flow, rising limb, and recession limb of the hydrograph components were accurately represented by the simulated and observed hydrographs, as illustrated in the aforementioned graph. Thus, the model's validation confirmed that the simulated hydrograph accurately depicts the observed hydrograph. The model underestimates the simulated discharge even though model performance indicated good agreement. This could be because of rainfall variance that is not accurately reflecting the area's rainfall, or it could be because of typing errors that cause the peak discharge to occur during the record of observed discharge.

### 3.2 Future projected climate change impact

#### 3.2.1 Change in future maximum temperature

**Fig. 8:**
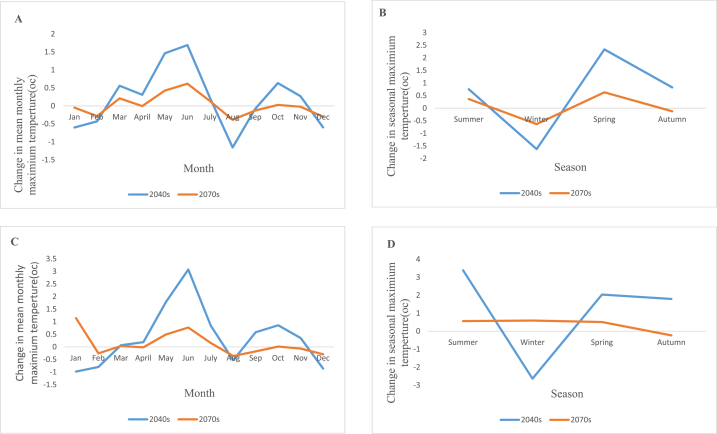
Change in mean monthly maximum temperature for RCP4.5 (A), change in seasonal maximum temperature for RCP4.5 (B), change in mean monthly maximum temperature for RCP8.5 (C) and change in seasonal maximum temperature for RCP8.5(D).

For the RCP4.5 scenario in the year 2040s, the mean seasonal maximum temperature rises between 0.2 °C and 2.3 °C, with the spring season seeing the largest increases, and falls between 0.08 °C and 1.6 °C, with the winter seeing the largest drops. And in the same scenario for the 2070s, the average seasonal maximum temperature rises between 0.1 °C and 0.6 °C with the greatest springtime increases and falls between 0.005 °C and 6.3 °C with the greatest wintertime drops. In the 2040s time frame under RCP8.5, the mean seasonal maximum temperature increases between 0.07°C and 3.4 °C, with the summer showing the largest increase, and decreases between 0.5 °C and 0.98 °C, with the January showing the largest decline. While in 2070s the mean seasonal maximum temperature rises between 0.04 °C and 1.1 °C with the highest increase seen in January and falls between 0.02 °C and 0.36 °C with the highest decline seen in August. Fig. 8(A,B,C and D) shows the change in mean monthly maximum temperature for RCP4.5, change in seasonal maximum temperature for RCP4.5, change in mean monthly maximum temperature for RCP8.5 and change in seasonal maximum temperature for RCP8.5 respectively.

#### 3.2.2 Change in future minimum temperature

**Fig. 9:**
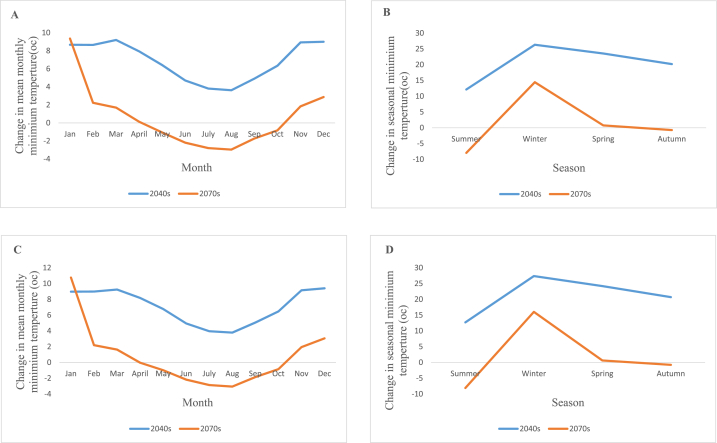
Change in mean monthly minimum temperature for RCP4.5 (A), change in mean seasonal minimum temperature for RCP4.5 (B), change in mean monthly minimum temperature for RCP 8.5(C) and change in mean seasonal minimum temperature for RCP8.5 (D).

The average monthly minimum temperature increases between 3.6 °C and 23.5 °C in the 2040s periods under the RCP4.5 scenario, with spring showing the largest increases and no discernible decreases. Additionally, during the 2070s, the average seasonal minimum temperature would fluctuate between 0.1 °C and 14.5 °C, with winter showing the largest increases, and 0.7 °C–3 °C, with August showing the largest declines. In the 2040s, under the RCP8.5 scenario, the mean seasonal minimum temperature rises between 3.8°C and 27.4 °C, with the greatest increases occurring in the winter and no discernible decreases. And in the 2070s, the average seasonal minimum temperature will increase between 0.6 °C and 16 °C with the largest increases occurring in the winter and decrease between 0.03 °C and 8.1 °C with the largest declines occurring in the summer. Fig. 9 (A,B,C and D) shows the change in mean monthly minimum temperature for RCP4.5, change in seasonal minimum temperature for RCP4.5, change in mean monthly minimum temperature for RCP 8.5 and change in seasonal minimum temperature for RCP8.5 scenario respectively.

#### 3.2.3 Change in future evapotranspiration

**Fig. 10:**
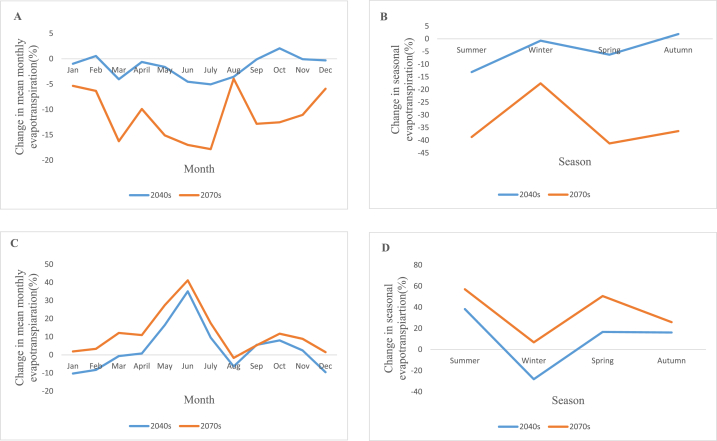
Change in average monthly evapotranspiration for RCP4.5 (A), change in seasonal evapotranspiration for RCP4.5 (B), change in mean monthly evapotranspiration RCP8.5(C) and change in seasonal evapotranspiration for RCP8.5 (D).

The average seasonal evapotranspiration increases in the 2040s periods under RCP4.5 scenarios in a range from 0.6% to 2.2% with highest increments in October and drops in a range from 0.1% to 5% with largest decrements in July. The average seasonal evapotranspiration declines between 3.9% and 17.8%in the 2070s periods of the RCP4.5 scenario, with maximum decreases occurring in July and no seasonal increases. The average seasonal evapotranspiration increases in the 2040s periods of the RCP8.5 scenarios in a range from 0.8% to 38.3%, with highest increases in the summer, and drops in a range from 0.6% to 28.1%, with largest decreases in the winter. For the 2070s of the RCP8.5, seasonal evapotranspiration rises between 1.5% and 57%, peaking in the summer and falling by 1.7% in August. Fig. 10 (A, B, C and D) shows the change in mean monthly evapotranspiration for RCP4.5, change in seasonal evapotranspiration for RCP4.5, change in mean monthly evapotranspiration for RCP8.5 and change in seasonal evapotranspiration for RCP8.5 respectively.

#### 3.2.4 Change in future rainfall

**Fig. 11:**
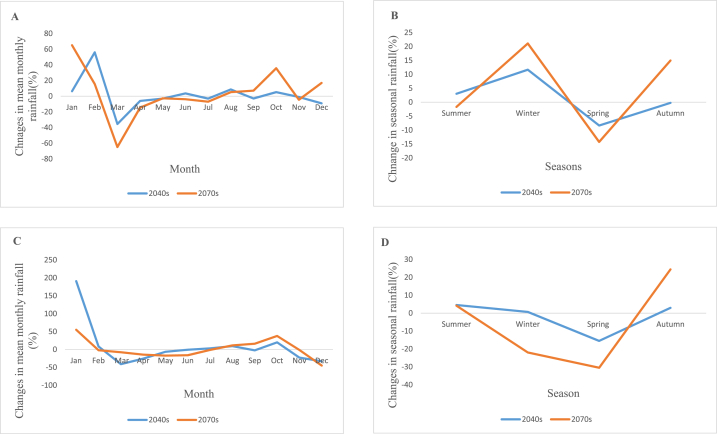
Change in average monthly rainfall for RCP4.5 (A), change in average seasonal rainfall for RCP4.5 (B), change in average monthly rainfall RCP8.5(C) and change in average seasonal rainfall for RCP8.5 (D).

The average seasonal rainfall increases in the 2040s periods under the RCP4.5 scenario in a range from 3% to 56%, with highest increases in February, and falls in a range from 1% to 35.5%, with maximum declines in March. Additionally, the average seasonal rainfall in the 2070s shows increases in the range of 5.2%–65.1%, with largest increases in January, and declines in the range of 1.7%–64.9%, with maximum drops in March. The average seasonal rainfall increases between 0.7% and 190.9% under the RCP8.5 scenario of the 2040s periods, with the largest increases occurring in January, and drops between 0.5% and 40.8%, with the largest decreases occurring in March. While rainfall in the 2070s grew from 11.6% to 55.2%, with the largest increases occurring in January, and fell from 0.4% to 44.8%, with the largest decreases occurring in December. Fig. 11(**A,B,C and D)** Depicts the change in average monthly rainfall for RCP4.5, change in seasonal rainfall for RCP4.5, change in mean monthly rainfall for RCP8.5 and change in seasonal rainfall for RCP8.5 respectively.

#### 3.2.5 Change in future flow

**Fig. 12:**
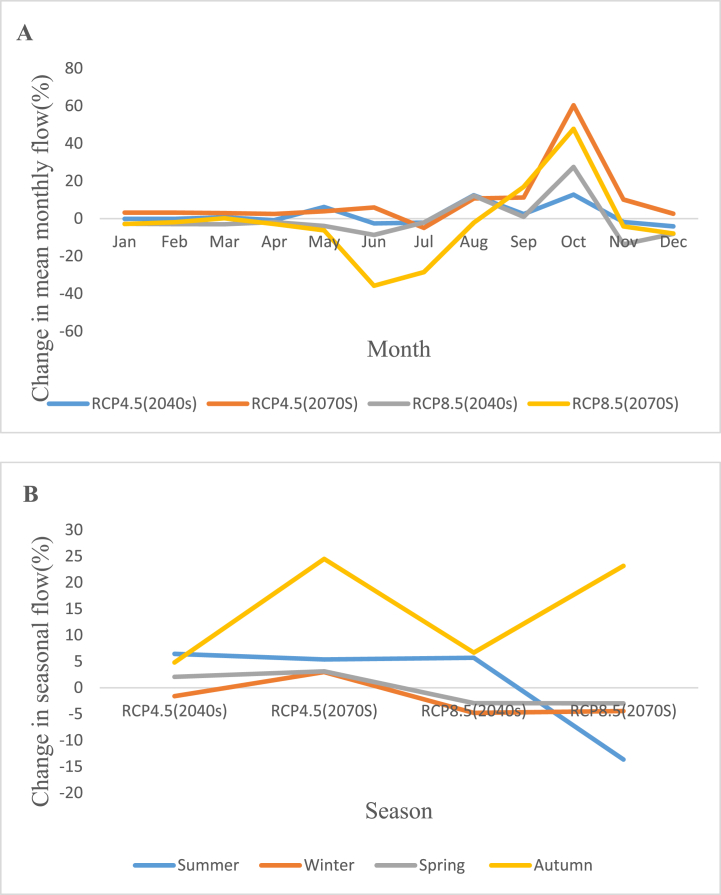
Change in average monthly flow for RCP4.5 and RCP8.5 (A) and change in average seasonal flow for RCP4.5 and RCP8.5 (B).

In the 2040s, according to the RCP4.5 scenario, the average seasonal flow varies from 0.7 to 12.5% with the largest rises occurring in August, and from 0.1 to 4.1% with the largest declines occurring in December. Additionally, during the 2070s, the seasonal flow average grows between 2.96% and 60.3% on average, reaching its highest levels in October and dropping by 5% in July. The average seasonal flow increases in a range from 0.9% to 27.5% with maximum increments in October and lowers in a range from 1.8% to 13.6% with highest decrements in November under the RCP8.5 scenario of the 2040s periods. In addition, under the same scenario in the 2070s, the average seasonal flow shows largest rises in October, a range of 0.2%–47.8%, and maximum declines in June, a range of 1.9%–35.8%. Fig. 12(A, and B) shows the change in average monthly flow for RCP4.5 and RCP8.5 and change in average seasonal flow for RCP4.5 and RCP8.5 respectively.

#### 3.2.6 Change in future water availability

Evapotranspiration has been estimated based on Enku's and melese methods [20] which is less data intensive method and applicable for data scarce regions. Water availability has been estimated with the formula mentioned in Ref. [21] as follows.

(6) W_am_ = P_m_ - ET_m_

Where W_am_ represents the amount of available water for a given month, P_m_ represents the amount of precipitation for a given month, and ET_m_ represents the potential evapotranspiration for a given month. After Equation (6) has been used to compute the water availability in the baseline and future scenarios, the difference in water availability between the two scenarios has been used to estimate the change in water availability.

**Fig. 13:**
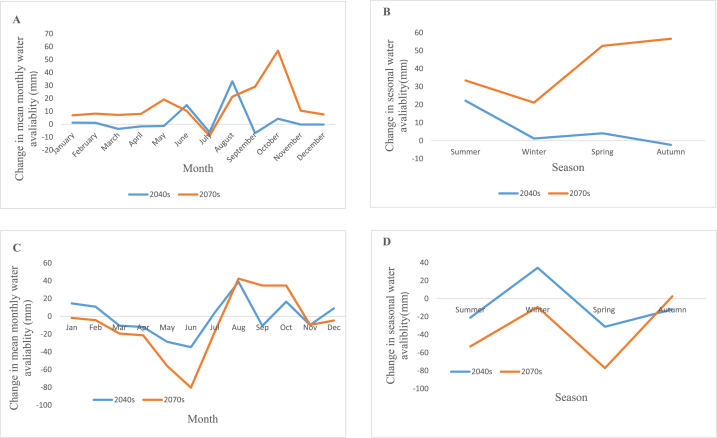
Change in average monthly water availability for RCP4.5 (A), change in average seasonal water availability for RCP4.5 (B), change in average monthly water availability RCP8.5(C) and change in average seasonal water availability for RCP8.5 (D).

In the periods of 2040s under RCP4.5 scenario, the average seasonal water availability shows neither an increasing nor decreasing trend. The increase in seasonal water availability ranges from 1.1 to 33.2 mm, with August showing the highest increase, while the decrease ranges from 0.23 to 6.89 mm, with September showing the highest decrease. And for the same scenario's periods in the 2070s, water availability rises between 7.2 mm and 56.9 mm, with the largest gains occurring in October and the smallest declines occurring in July (by 9 mm). The average seasonal water availability in the 2040s under RCP8.5 improves between 4.1 mm and 38.8 mm with a greatest increase in August and drops between 9.8 mm and 31.2 mm with a maximum decline in the spring seasons. For the 2070s under RCP8.5 water, availability improves between 2.7 mm and 42.4 mm with highest increments in August and displays decrements between 1.8 mm and 80.3 mm with maximum decrements in June. The outcome showed that both scenarios and subsequent times have lower water availability during the dry season. . Under the two scenarios and future times, water availability in the watershed improves during the wet season. The future dry season's reduced water supply may have an influence on irrigation production, which further reduces society's ability to provide for its own food needs. Under RCP8.5, which represents the worst case scenario, the impact of climate change on this watershed's water resource availability is probably greater than it would be under RCP4.5. Fig. 13 (A, B, C, and D) shows the change in average monthly water availability for RCP4.5, change in average seasonal water availability for RCP4.5, change in average monthly water availability for RCP8.5, and change in average seasonal water availability for RCP8.5 respectively.

## 4 Discussions

The findings demonstrated that the Kiltie watershed's water resource availability is significantly impacted by climate change. Water availability improves during the wet seasons and may decrease during the dry seasons in the 2040s and 2070s periods under RCP4.5 and RCP8.5. A decrease in rainfall, flow, and an increase in evapotranspiration could all contribute to the dry season's decreased water supply. While the increase in water availability during the rainy seasons may result from higher rainfall, increased flow, and decreased evapotranspiration. This study confirms findings of [22,23] that flow in the gauged Lake Tana watersheds could drop during the dry season and increase during the wet season under RCP4.5 and RCP8.5 scenarios for the 2040s and 2070s periods. Additionally, the outcome differed from Ref. [24] research, which showed that for the A1B scenario, rainfall, flow, and water resource availability increase in the Upper Blue Nile Basin during the wet and dry seasons. This study reveals dry season water availability declines and rainy season water availability increases, which is in contrast to other studies by Ref. [25] in the Dong Nai River Basin in Vietnam that show dry season water availability increases. Due to the use of various GCM-RCM models and climate scenarios with vastly different levels of resolution and emission rate, the results of this study and previous ones have varied. These studies have used fine resolution GCM-RCM models and the latest representative concentration pathway scenarios (RCPs) as compared to special report emission scenarios (SRES) which is used by previous studies. In climate change study, uncertainty sources can be acquired from downscaling method, GCMs, scenarios and the hydrological models used. According to Refs. [26,27] most of the uncertainties arise from GCM models used, and recommends to use multiple GCMs for reducing wrong conclusions due to uncertainty. Hence, this study used an ensemble of two GCMs which supports the use of multiple GCMs can reduce uncertainty. Unlike previous studies on this basin, this study has a unique feature of using ensemble GCMs, the latest RCP scenarios, and the HBV hydrological model (applicable for data scare regions) that can reduce uncertainty as compared with previous studies that are done using single GCMs and an outdated scenario (SRES). The majority of earlier research in the Lake Tana Basin employed a coarse resolution GCM and outdated scenarios to examine the effects of climate change on flow. Unlike other studies, this study is focused on water resource availability considering loss (potential evaporation) with fine-resolution GCMs and the latest RCP scenarios.

## 5 Conclusions

According to the study, climate change will have a significant impact on the availability of water resources in the upper Blue Nile basin of the Kiltie watershed. Using ensemble climate models with one regional climate model and one hydrological model, changes in temperature, evapotranspiration, rainfall, flow, and water availability in the Kiltie watershed under two future scenarios (RCP4.5 and RCP8.5) were assessed. For all RCP scenarios and future periods relative to the baseline periods, the modeling result demonstrates that temperature rises in the dry seasons and falls in the rainy seasons. For all RCP4.5 and RCP8.5 of the 2040s and 2070s periods, future evapotranspiration will rise during the dry season and fall during the rainy season. In the future RCP4.5 and RCP8.5 scenarios for the 2040s and 2070s periods, rainfall, flow, and water availability show increases in wet seasons and decreases in dry seasons where irrigation practice is practiced. The study is the first of its sort and uses the most recent scenarios, GCM, and RCM models. The study found that future water availability in watersheds where irrigation is performed during the dry season could be decreased, which would promote the adoption of water-stress crops. This study indicates that future increments in water availability and flow in the wet season helps to accommodate excess storage for dry season irrigation while future flow and water availability decrements show there will have a scarcity of water for dry season irrigation that requires coordinated water and watershed management, as well as a future watershed-level development plan.

## Acknowledgments

We sincerely thank all organizations and individuals that provided information for our study. The relevant information has been gathered by the government entities West Amhara Meteorological Agency, Abay Basin Authority, and Ministry of Water, Irrigation, and Energy.

## Author contribution statement

Melsew A. Wubneh, Tadege A. Worku: Conceived and designed the analysis; Analyzed and interpreted the data; Contributed analysis tools or data.

Bantalem Z. chekol: Contributed analysis tools or data; Wrote the paper.

## Funding statement

This research did not receive any specific grant from funding agencies in the public, commercial, or not-for-profit sectors.

## Data availability statement

Data will be made available on request.

## Declaration of interest's statement

The authors declare no conflict of interest.

## References

[1] Wubneh M.A., Worku T.A., Fikadie F.T., Aman T.F., Kifelew M.S. (2022). Climate change impact on Lake Tana water storage, upper Blue Nile Basin, Ethiopia. Geocarto Int..

[2] Pervez M.S., Henebry G.M. (2015). Assessing the impacts of climate and land use and land cover change on the freshwater availability in the Brahmaputra River basin. J. Hydrol. Reg. Stud..

[3] Azari M., Moradi H.R., Saghafian B., Faramarzi M. (2016). Climate change impacts on streamflow and sediment yield in the North of Iran. Hydrol. Sci. J..

[4] Kofi J., Ofosu E.A., Akpoti K., Kabo-bah A.T. (2022). Modeling current and future groundwater demands in the white volta River Basin of Ghana under climate change and socio-economic scenarios journal of hydrology : regional studies modeling current and future groundwater demands in the white volta river basi. J. Hydrol. Reg. Stud..

[5] Obahoundje S., Ofosu E.A., Akpoti K., Kabo-bah A.T. (2017). Land use and land cover changes under climate uncertainty: modelling the impacts on hydropower production in Western Africa. Hydrology.

[6] Zegeye H. (2018). Climate change in Ethiopia : impacts. Mitigation and Adaptation.

[7] Setegn S.G., Rayner D., Melesse A.M., Dargahi B., Srinivasan R. (2011). Impact of climate change on the hydroclimatology of Lake Tana basin, Ethiopia. Water Resour. Res..

[8] WaleWorqlul A., Taddele Y.D., Ayana E.K., Jeong J., Adem A.A., Gerik T. (2018). Impact of climate change on streamflow hydrology in headwater catchments of the upper Blue Nile Basin, Ethiopia. Water (Switzerland).

[9] Ayele H.S., Li M.H., Tung C.P., Liu T.M. (2016). Impact of climate change on runoff in the Gilgel abbay watershed, the upper Blue Nile Basin, Ethiopia. Water (Switzerland).

[10] Teklay A., Dile Y.T., Asfaw D.H., Bayabil H.K., Sisay K. (2021). Impacts of climate and land use change on hydrological response in Gumara watershed, Ethiopia. Ecohydrol. Hydrobiol..

[11] Mengistu D., Bewket W., Dosio A., Panitz H.J. (2021). Climate change impacts on water resources in the upper Blue nile (Abay) River Basin, Ethiopia. J. Hydrol..

[12] Abebe E., Kebede A. (2017). Assessment of climate change impacts on the water resources of megech river catchment, abbay basin, Ethiopia. Open J. Mod. Hydrol..

[13] Dile Y.T., Berndtsson R., Setegn S.G. (2013). Hydrological response to climate change for Gilgel Abay River, in the Lake Tana basin - upper Blue Nile Basin of Ethiopia. PLoS One.

[14] Bokke A.S., Taye M.T., Willems P., Siyoum S.A. (2017). Validation of general climate models (GCMs) over upper Blue nile River Basin, Ethiopia. Atmos. Clim. Sci..

[15] Yeboah K.A. (2022). Assessing climate change projections in the Volta Basin using the CORDEX-Africa climate simulations and statistical bias-correction. Environ. Challenges.

[16] Ringard J., Seyler F., Linguet L. (2017). A quantile mapping bias correction method based on hydroclimatic classification of the Guiana shield. Sensors.

[17] Fang G.H., Yang J., Chen Y.N., Zammit C. (2015).

[18] Toum E., Masiokas M.H., Villalba R., Pitte P., Ruiz L. (2021). The HBV. IANIGLA Hydrological Model.

[19] Abdo K.S., Fiseha B.M., Rientjes T.H.M., Gieske A.S.M., Haile A.T. (2009). Assessment of climate change impacts on the hydrology of Gilgel Abay catchment in Lake Tana basin, Ethiopia. Hydrol. Process..

[20] Enku T., Melesse A.M. (2013). A simple temperature method for the estimation of evapotranspiration Temesgen. Hydrol. Process..

[21] Taye M.T., Dyer E., Hirpa F.A., Charles K. (2018). Climate change impact on water resources in the Awash basin, Ethiopia. Water (Switzerland).

[22] Wubneh M.A., Fikadie F.T., Worku T.A., Aman T.F., Kifelew M.S. (2022). Hydrological impacts of climate change in gauged sub-watersheds of Lake Tana sub-basin (Gilgel Abay, Gumara, megech, and ribb) watersheds, upper Blue Nile Basin, Ethiopia. Sustain. Water Resour. Manag..

[23] Wubneh M.A., Kifelew M.S., Sahlu D., Dzwairo R.B., Fikadie F.T. (2022). Hydrological impacts of climate change in selected ungauged sub-watersheds of Lake Tana Sub-Basin, Upper Blue Nile Basin, Ethiopia: a regionalization approach. Sci. African.

[24] Roth V., Lemann T., Zeleke G., Teklay A. (2018). Effects of climate change on water resources in the upper Blue Nile Basin of Ethiopia. Heliyon.

[25] Khoi D.N., Nguyen V.T., Sam T.T., Mai N.T.H., Vuong N.D., Cuong H.V. (2021). Assessment of climate change impact on water availability in the upper Dong Nai River Basin, Vietnam. J. Water Clim. Chang..

[26] Khoi D.N., Suetsugi T. (2012). Uncertainty in climate change impacts on streamflow in Be River Catchment, Vietnam. Water Environ. J..

[27] Hoan N.X., Khoi D.N., Thi P., Nhi T. (2018). Uncertainty assessment of streamflow projection under the impact of climate change in the Lower Mekong.

